# Identification of Reference Genes for qRT-PCR Analysis in Yesso Scallop *Patinopecten* *yessoensis*

**DOI:** 10.1371/journal.pone.0075609

**Published:** 2013-09-19

**Authors:** Liying Feng, Qian Yu, Xue Li, Xianhui Ning, Jing Wang, Jiajun Zou, Lingling Zhang, Shi Wang, Jingjie Hu, Xiaoli Hu, Zhenmin Bao

**Affiliations:** Key Laboratory of Marine Genetics and Breeding (MGB), Ministry of Education, College of Marine Life Sciences, Ocean University of China, Qingdao, China; Swedish University of Agricultural Sciences, Sweden

## Abstract

**Background:**

Bivalves comprise around 30,000 extant species and have received much attention for their importance in ecosystems, aquaculture and evolutionary studies. Despite the increasing application of real-time quantitative reverse transcription PCR (qRT-PCR) in gene expression studies on bivalve species, little research has been conducted on reference gene selection which is critical for reliable and accurate qRT-PCR analysis. For scallops, systematic evaluation of reference genes that can be used among tissues or embryo/larva stages is lacking, and β-actin (*ACT*) is most frequently used as qRT-PCR reference gene without validation.

**Results:**

In this study, 12 commonly used candidate reference genes were selected from the transcriptome data of Yesso scallop (* Patinopecten yessoensis *) for suitable qRT-PCR reference genes identification. The expression of these genes in 36 tissue samples and 15 embryo/larva samples under normal physiological conditions was examined by qRT-PCR, and their expression stabilities were evaluated using three statistic algorithms, geNorm, NormFinder, and comparative ∆Ct method. Similar results were obtained by the three approaches for the most and the least stably expressed genes. Final comprehensive ranking for the 12 genes combing the results from the three programs showed that, for different tissues, DEAD-box RNA helicase (*HELI*), ubiquitin (*UBQ*), and 60S ribosomal protein L16 (*RPL16*) were the optimal reference genes combination, while for different embryo/larva stages, gene set containing Cytochrome B (CB), Cytochrome C (CC), Histone H3.3 (*His3.3*), and Glyceraldehyde-3-phosphate dehydrogenase (GAPDH) were recommended for qRT-PCR normalization. *ACT* was among the least stable genes for both adult tissues and embryos/larvae.

**Conclusions:**

This work constitutes the first systematic analysis on reference genes selection for qRT-PCR normalization in scallop under normal conditions. The suitable reference genes we recommended will be useful for the identification of genes related to biological processes in Yesso scallop, and also in the reference gene selection for other scallop or bivalve species.

## Introduction

Gene expression studies are increasingly important in identifying genes, pathways, and networks underlying cellular and developmental processes. Real-time quantitative reverse transcription PCR (qRT-PCR) is frequently used technique for gene expression analysis, due to its advantage in sensitivity, speed, throughput, and speciﬁcity [1-3]. To ensure the reliability and accuracy of qRT-PCR analysis, reference gene(s) are needed to normalize the differences among samples and among PCR runs, including variations in amount of starting material, RNA extraction, and reverse transcription efficiency, etc [4,5]. The selection of reference gene(s), therefore, is critical for the accuracy of qRT-PCR analysis.

Ideally, reference genes used in qRT-PCR should be expressed constantly across various biological or experimental conditions, such as different tissues, developmental stages, or experimental treatments [6-9]. So they are usually chosen from housekeeping genes [10-12]. However, many reports showed that, housekeeping gene expression, although occasionally exhibits constancy in some given cell types or developmental stages, can fluctuate considerably [1,13,14]. Recent studies further demonstrated that the expression of classic reference genes, such as β-actin (*ACT*) [15-18] and glyceraldehyde-3-phosphate (*GAPDH*) [15,19,20], can vary extensively and are consequently unsuitable for normalization in gene transcription analysis on some species or samples. For particular species, biological conditions, and experimental designs, the utility of reference genes must be validated for the purpose of getting reliable qRT-PCR results [21].

Bivalves comprise around 30,000 extant species. Recently, for the importance of this class of invertebrates in ecosystems, aquaculture and evolutionary studies [22], more and more bivalve genes were discovered by genome sequencing [23,24], transcriptome sequencing [25-30], and cloning [31-34] etc., providing rich gene resources to the scientific community for the identification of differentially expressed genes related to functional traits. qRT-PCR had been frequently used in the expression analysis of bivalve genes, however, very few studies has been carried out on reference gene selection in these animals, and generally used reference genes in most species have not been validated. For example, in scallops, the most frequently used reference gene in qRT-PCR is *ACT*, but without evaluation. By far, in the limited studies on reference gene selection for bivalve species, most of them focused on single tissues, such as haemocyte in parasite attacked flat oyster ( *Ostrea* *edulis* ) [18] and bacteria infected soft-shell clam (*Mya* *Arenaria* ) [17], and gonad in mussel ( *Mytilus* *edulis* ) [35] and lion’s paw scallop ( *Nodipectensubnodosus* ) [36]. Although the expression stability of candidate reference genes was assessed in several tissues of Yesso scallop ( *Patinopecten* *yessoensis* ) with respect to starvation treatment, the reference genes were recommended for different single tissues, and those could be generally used among tissues were not evaluated or identified [37]. Therefore, the application of these selected reference genes is largely restricted to particular tissues or experimental conditions. Meanwhile, gene expression variations during bivalve development, which play critical roles in the tremendous morphology changes across embryo/larva stages, have been extensively studied [29,38-40], while related reference gene evaluation was only reported in oyster [41]. On the other hand, the number of candidate reference genes evaluated in single bivalve species is mostly no more than six [18,35,36] mainly due to the previously limited availability of gene sequences, thus increased the chances of missing more stably expressed genes.

Yesso scallop which naturally distributes along the coastline of northern Japan, the Far East of Russian, and the northern Korean Peninsula, has become one of the main maricultural shellfish in the north of China since it was introduced in 1982 [42]. For the economic and ecological importance of this species, the first large scale transcriptome sequencing for scallops was performed recently in *P* *. yessoensis* , and over 20,000 genes were obtained [25], providing massive gene resource for gene expression studies and systematic reference gene validation in this bivalve species. Herein, from the transcriptome data, we selected 12 frequently used reference genes, and evaluated their expression stabilities in different adult tissues and embryo/larva stages under normal physiological conditions, using three different algorithms which are commonly applied in reference gene assessment. The suitable reference genes recommended in this study could be generally used for qRT-PCR analysis in normal Yesso scallop, which will aid the identification of genes related to and in turn our understanding of biological processes in scallop.

## Materials and Methods

### Scallop Collection

All the experiments on scallops were conducted following the institutional and national guidelines. The Yesso scallop samples used in this study were collected from Zhangzidao Fishery Group Co., Dalian, China. Tissues from six healthy adult Yesso scallops (65.6 ± 1.7 mm in shell height), including mantle, gill, gonad, kidney, striated muscle and digestive gland were dissected, immediately frozen in liquid nitrogen, and then stored at -80°C. Normal embryos and larvae of Yesso scallop, including fertilized eggs, blastulae, gastrulae, trochophore larvae, and D-shaped larvae were collected and preserved at -80°C. For each of the above embryo/larva stage, 3 sets of samples (each set, n > 500) were collected. Thus, a total of 36 tissue samples and 15 embryo/larva samples were used in the following analysis.

### RNA Extraction and Reverse Transcription

Total RNA was isolated from the tissues and embryos/larvae using traditional RNA isolation methods described by Hu et al. [43], and then digested with DNase I (Takara Bio, Shiga, Japan) to eliminate potential DNA contamination. The integrity of the RNA samples was checked by agarose gel electrophoresis. RNA concentration and purity were detected using Nanovue Plus (GE healthcare, Milwaukee, USA). Only RNA samples with clear bands corresponding to 28S and 18S rRNA on the gel, OD_260_/OD_280_ ratio between 1.8 and 2.0, and OD_260_/OD_230_ ratio higher than 2.0 were used for subsequent analysis. Each RNA sample was assayed in triplicate and the average value was determined. The first-strand cDNA was synthesized according to the manufacturer’s instruction of M-MLV Reverse Transcriptase (Promega, Wisconsin, USA) with 2µg DNase I-treated total RNA and 2µM Oligo (dT)_18_ (Takara Bio) primer in a 25-µL volume (Table 1). A control reaction without reverse transcriptase (no-RT control) was performed to preclude any DNA contamination through the subsequent PCR analysis.

**Table 1:** Information of the 12 candidate reference genes and primers used in this study.

**Gene symbol**	**Gene name**	**Gene function**	**Primer Sequence (5’–3’**)	**Product size (bp**)	**Efficiency**
CT	β-actin	Cytoskeleton	CCAAAGCCAACAGGGAAAAG	163	96.87%
			TAGATGGGGACGGTGTGAGTG		
GAPDH	Glyceraldehyde-3- phoshate ehydrogenase	Glycolysis enzyme	GGTATGGCTTTCCGTGTGC	193	99.27%
			TGCTGCTTCTTGCGTCTCC		
CC	Cytochrome C	Electron transportation	CGTTTTCTCCTGGTTCTTCGTC	178	97.51%
			TCTTCCTCTCCACCCTTTCTAGTC		
CB	Cytochrome B	component of respiratory chain complex III	CCTCTCCACCCTTTCTAGTCCTTG	170	96.86%
			CTCCTGGTTCTTCGTCTTTCTCC		
EF-1-β	elongation factor 1-β	Translation	CAGTTTCCAAGGCTCCCAAT	140	99.10%
			AGCGTCTCCTGAAGGTCCAT		
UBQ	Ubiquitin	Protein degradation	TCGCTGTAGTCTCCAGGATTGC	184	99.28%
			TCGCCACATACCCTCCCAC		
TBP	TATA-box binding protein	RNA polymerase transcription factor	AGTCTACACTTGCTGCTGAACTTTG	170	98.62%
			CCTTGGCCCATCTTCTCCTC		
RPL16	60S ribosomal protein L16	Ribosome Protein	CTGCCAGACAGACTGAATGATGCC	117	96.86%
			ACGCTCGTCACTGACTTGATAAACCT		
HELI	DEAD-box RNA helicase	RNA unwinding	CCAGGAGCAGAGGGAGTTCG	186	98.66%
			GTCTTACCAGCCCGTCCAGTTC		
TUB	β-Tubulin	Cytoskeleton	CCTGGGTTCGCTCCTCTCAC	112	99.20%
			ACATAGCAGCAACTGTCAGATAACG		
CYP	Cyclophilin A	Immune- suppression	AGATGCTCTTTCCACCAGTTCCA	165	98.63%
			TGCGTGCTGATGTTGTGCCTA		
His3.3	Histon H3.3	DNA strands compaction	TAGTATGACTTGCATGATCCGTAGAAA	121	98.34%
			GCCAGAAGAATCCGTGGTGAA		

### Candidate reference genes selection

The transcriptome data of Yesso scallop (accession number: SRA027310) was analyzed to search for the orthologs of candidate reference genes previously reported in qRT-PCR analysis. The assembled transcriptome sequences were compared against the NCBI non-redundant (Nr) protein database and Swiss-Prot database using BlastX [25]. Gene name was initially assigned to each assembled sequence based on the best BLAST hit. Then the protein sequences of the best BLAST hits were further performed tBlastn against the Yesso scallop transcriptome. Assembled sequence which could be annotated as the same protein again with best hit was used for candidate reference gene searching. Finally, a total of 12 commonly used candidate reference genes [18,35,44-47], including *ACT*, *GAPDH*, Cytochrome C (CC), Cytochrome B (CB), elongation factor 1-β (*EF-1-β*), Ubiquitin (*UBQ*), TATA-box binding protein (TBP), 60S ribosomal protein L16 (*RPL16*), DEAD-box RNA helicase (*HELI*), β-Tubulin (*TUB*), Cyclophilin A (*CYP*), and Histone H3.3 (*His3.3*) were selected for expression stability analysis (Table 1).

### Primer design and qRT-PCR

Primers for qRT-PCR were designed using the Primer 5.0 software (http://www.premierbiosoft.com/primerdesign/index.html). The sizes of PCR products were between 100-200 bp. The annealing temperature for all primer-pairs was optimized to 62.8°C. Amplicons of each primer pair were tested by 2% agarose gel electrophoresis to verify the products size and specificity. Then the target amplicons were purified using MinElute Gel Extraction Kit (Qiagen, Düsseldorf, Germany), and sequenced in Sangon Biotech (Shanghai, China) to confirm the sequence specificity. For each primer pair, amplification products of no-RT control were run to rule out potential DNA contamination during RNA isolation, and no-template control amplification was performed to ensure the absence of other contamination or primer dimer. The information of the primers is listed in Table 1.

qRT-PCR reactions were conducted in a 96-well plate using ABI7500 Real-Time system (Applied Biosystems, CA, USA). Each reaction was performed in triplicate and in a 20-µL volume containing 1× Real-time PCR Master Mix with SYBR Green dye (TOYOBO, Osaka, Japan), 0.4 µM of each primer and 2 µL cDNA, using the following thermal conditions: 50°C for 2min, 95°C for 10min, followed by 40 cycles of 95°C for 15s and 62.8°C for 1min. A melting curve analysis (60 °C to 95 °C) was performed at the end of each PCR to further confirm the specificity of amplicons. The raw fluorescence data were exported and analyzed using an online software Real-time PCR Miner (http://www.miner.ewindup.info/miner/Version2) [48], and the threshold cycle (Ct) value for each reaction and amplification efficiency of each gene was calculated and provided by this software.

### Analysis of gene expression stability

The expression stability of the selected reference genes was evaluated using three frequently used statistical approaches [18,19,49,50], geNorm V3.5 (http://medgen.ugent.be/jvdesomp/geNorm/) [51], NormFinder (http://www.mdl.dk/publicationsnormfinder.htm) [52], and comparative ΔCt method [53] to get complementary assessments.

GeNorm is a popular algorithm to determine the most stable reference genes from the candidate ones, and the optimal number of genes needed for accurate normalization in qRT-PCR [51]. After transforming the raw Ct values into relative quantification data, this software calculates the value of gene expression stability (M) for all candidate genes, based on the assumption that, expression ratio of the ideal reference genes should be stable among the samples tested. The M value is defined as the pairwise variation of a reference gene with all other reference genes. To get a rank, gene with the highest M value (the worst reference gene) is eliminated and the new M values for the other candidate genes are recalculated, until only two genes are remained as the most stable ones. For the minimum number of genes used to get accurate result, geNorm calculates the pairwise variation Vn/Vn+1 which represents the variation between using n most stable genes and using n+1 most stable genes. This evaluation uses 0.150 as the cut-off, below which the inclusion of an additional reference gene is not required, that is, n reference genes is sufficient for accurate normalization.

NormFinder is a model-based approach for identifying stable reference genes through calculating intra- and intergroup variations which are then combined into a stability value [52]. Candidate reference genes with the least intra- and intergroup variation, therefore the least stability value, are considered to be stable with top rank. This program could decrease the effect of correlated expression of the candidate reference genes.

The comparative ΔCt method was used to estimate the gene expression stability further. By comparing relative expression of gene pairs within each sample for all possible gene combinations, this method calculated the mean of standard deviation for each candidate genes [53]. The expression stabilities of the candidates were ranked according to their mean standard deviation, and the gene with the lowest standard deviation was identified as the most stable one.

## Results

### Primer specificity and amplification efficiency

The performance of the primer pairs were validated for both amplification specificity and efficiency. Agarose gel electrophoresis showed that, the amplification products of each primer pair appeared as a single band with the expected size. Then the PCR products were further confirmed by sequencing, and the specificity of the products from each primer pair were also validated as only a single peak was present in melting curve analysis. Amplification efficiency of all the primer pairs varied from 96.86% for *CB* to 99.28% for *UBQ* (Table 1). So the primers designed were acceptable for further qRT-PCR assays.

### Expression profile of the candidate reference genes in scallop tissues

**Figure 1:**
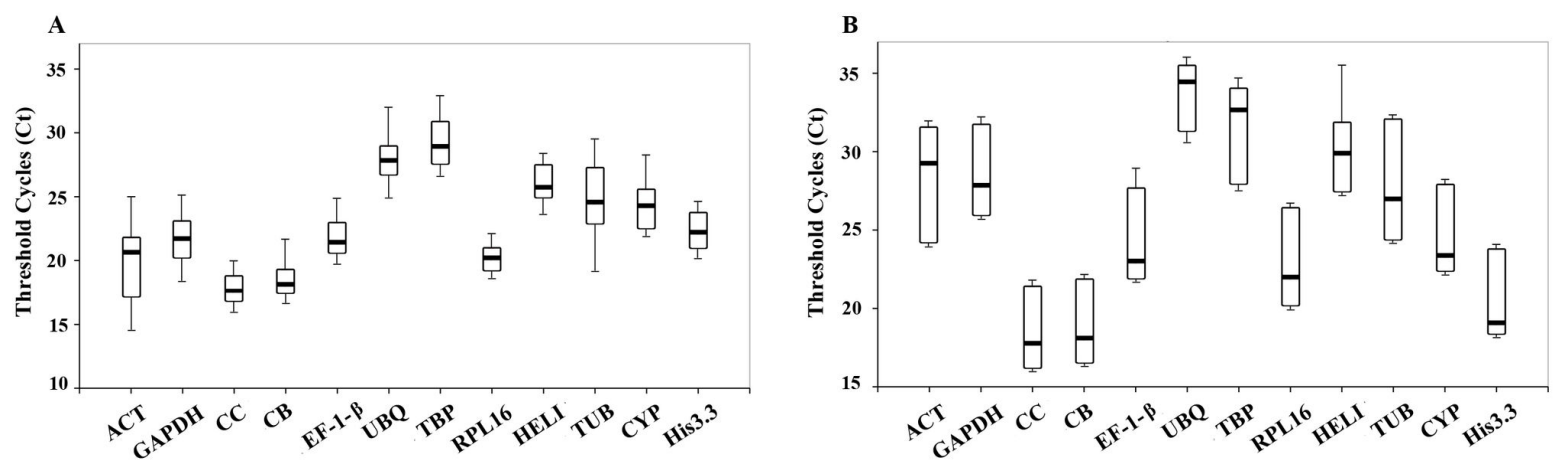
Threshold cycle (Ct) values for 12 candidate reference genes obtained using qRT-PCR in Yesso scallop tissues (A) and embryos/larvae (B). Box shows the 25/75 percentile. A line across the box indicates the median. Whisker caps represent the maximum and minimum values.

In all the tissue samples tested, the Ct values of the 12 genes ranged from 14.6 to 31.1 (Table S1). As shown in Figure 1A, the most highly expressed genes were *CC* and *CB*, which exhibited a median Ct value of 17.7 and 18.2, respectively. All other genes had median Ct values larger than 20, and *TBP* presented the lowest level of expression with the median Ct value as high as 29.0. In addition, the ranges of the Ct values for different tissues showed considerable variation among the 12 candidate genes (Table S1). The lowest ranges of Ct value were exhibited by *RPL16* and *CC*, indicating they are more stably expressed than other genes in scallop tissues. *ACT* and *TUB* were the genes showed higher Ct ranges than others, thus their expression levels might be more affected by tissue types. However, for expression stability evaluation, simple comparison of the raw Ct values for the candidate reference genes could not provide sufficient information. So the following analysis was conducted using three different statistical algorithms for reference genes validation.

### Expression stability of the candidate reference genes in scallop tissues

**Figure 2:**
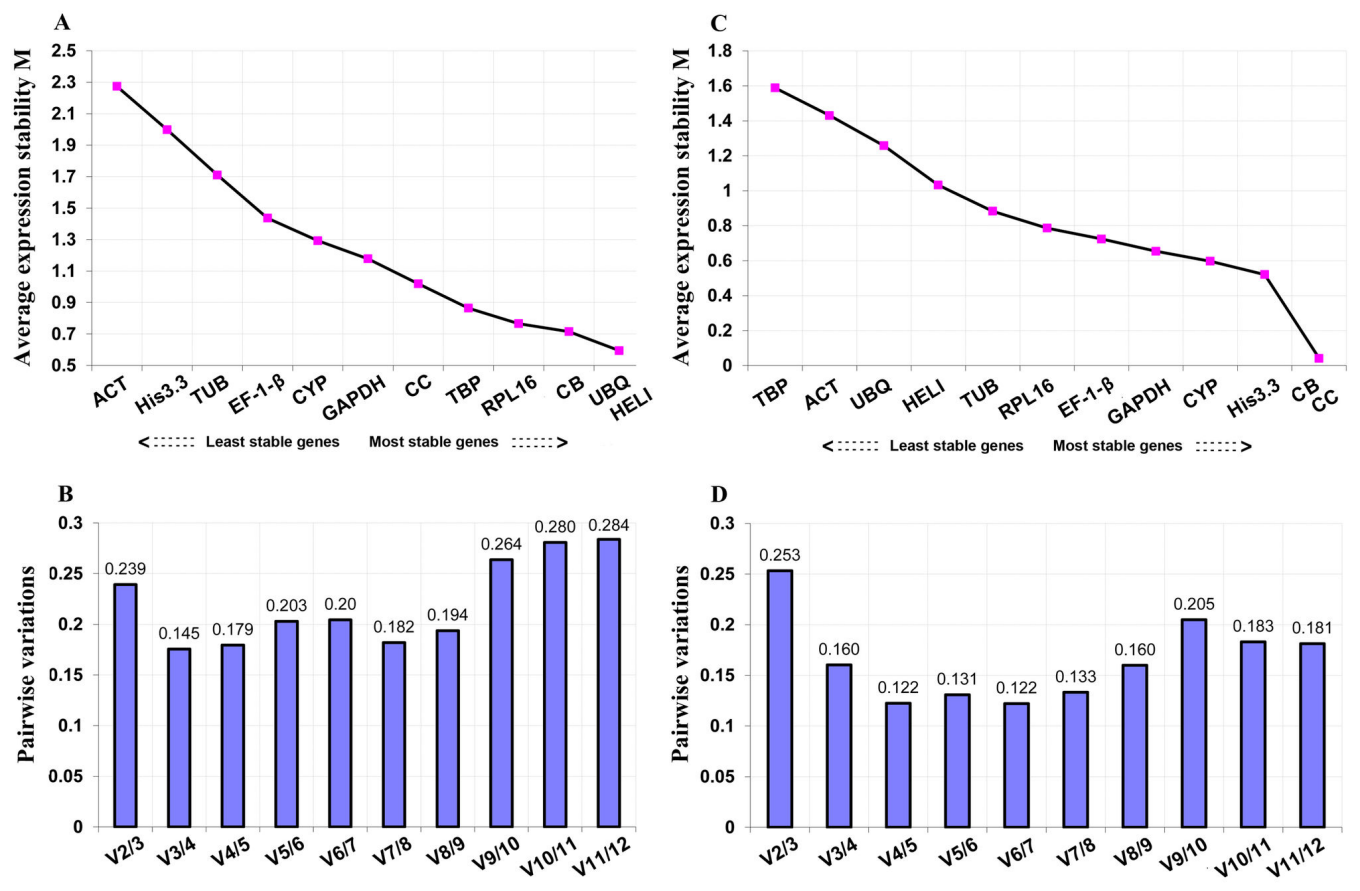
Expression stability of 12 candidate reference genes analyzed using geNorm for qRT-PCR in Yesso scallop tissues (A, B) and embryos/larvae (C, D). The expression stability (M) of each gene was evaluated during stepwise exclusion of the least stable gene (A, C). The optimal number of reference genes required for accurate normalization was determined through calculating pairwise variation Vn/Vn+1(B, D).

The expression level data of these candidate reference genes were analyzed using three algorithms, geNorm, NormFinder, and the comparative ∆Ct method. GeNorm ranked the candidate genes based on their average expression stability (M values), and a lower M value indicated more stable expression. For different tissues, geNorm analysis showed that, *HELI* and *UBQ* were the most stable genes, which were followed by *CB*, *RPL16*, *TBP*, *CC*, *GAPDH*, *CYP*, *EF-1-β*, *TUB*, *His3.3*, and *ACT* (Figure 2A, Table 2). Except *TUB, His3.3*, and *ACT*, all the candidate genes exhibited M values less than 1.50, the default cut-off value suggested by geNorm. The optimal number of genes required for accurate normalization in qRT-PCR was also presented by geNorm, through calculating pairwise variation Vn/Vn+1 which uses 0.150 as the proposed cut-off value. A Vn/Vn+1 less than 0.150 means that the top n reference genes are adequate for accurate qRT-PCR normalization. Here the V3/V4 value was 0.145 (Figure 2B), thus the top three reference genes (*HELI, UBQ*, and *CB*) would be adequate in qRT-PCR normalization for scallop tissues, and the addition of the forth gene is not necessary.

**Table 2:** Ranks of the candidate reference genes for qRT-PCR analysis in Yesso scallop tissues and embryos/larvae.

	**Rank**	**geNorm**	**NormFinder**	**Comparative ΔCt method**	**Comprehensive rank**
**Tissues**	1	HELI (0.595)	HELI (0.292)	HELI (1.274)	HELI
	2	UBQ (0.595)	UBQ (0.521)	RPL16 (1.427)	UBQ
	3	CB (0.715)	CB (0.557)	UBQ (1.472)	RPL16
	4	RPL16 (0.765)	RPL16 (0.59)	CB (1.48)	CB
	5	TBP (0.864)	GAPDH (0.634)	CC (1.689)	CC
	6	CC (1.019)	TBP (0.719)	GAPDH (1.717)	GAPDH
	7	GAPDH (1.177)	CC (0.834)	CYP (1.752)	TBP
	8	CYP (1.293)	EF-1-β(0.874)	TBP (1.775)	CYP
	9	EF-1-β(1.436)	CYP (0.95)	EF-1-β(1.911)	EF-1-β
	10	TUB (1.701)	His3.3 (1.321)	TUB (2.372)	TUB
	11	His3.3 (1.997)	TUB (1.568)	His3.3 (2.582)	His3.3
	12	ACT (2.274)	ACT (2.057)	ACT (2.899)	ACT
**Embryos/larvae**	1	CB (0.041)	CB (0.237)	CB (1.114)	CB
	2	CC (0.041)	CC (0.243)	CC (1.118)	CC
	3	His3.3 (0.52)	His3.3 (0.448)	His3.3 (1.172)	His3.3
	4	CYP (0.597)	RPL16 (0.531)	GAPDH (1.186)	GAPDH
	5	GAPDH (0.654)	EF-1-β(0.537)	CYP (1.211)	CYP
	6	EF-1-β(0.724)	GAPDH (0.540)	EF-1-β(1.249)	EF-1-β
	7	RPL16 (0.786)	CYP (0.549)	RPL16 (1.356)	RPL16
	8	TUB (0.883)	TUB (0.605)	TUB (1.356)	TUB
	9	HELI (1.032)	HELI (0.900)	HELI (1.490)	HELI
	10	UBQ (1.258)	UBQ (1.175)	ACT (1.906)	UBQ
	11	ACT (1.430)	TBP (1.234)	UBQ (2.063)	ACT
	12	TBP (1.588)	ACT (1.301)	TBP (2.068)	TBP

Note: The candidate reference genes were ordered from the most to the least stable, based on their stability values (in brackets) calculated by different algorithms. Lower stability value means more stable expression. The comprehensive rank for each gene was based on the geometric mean of its ranks from geNorm, NormFinder, and comparative ΔCt method, and gene with smaller value of geometric mean was ranked as more stable.

Then the expression data were analyzed with NormFinder. This program evaluates the stabilities of reference genes through calculating intra- and inter-group variations. From the most to the least stable, NormFinder ranked the 12 candidate genes as follows: *HELI*, *UBQ*, *CB*, *RPL16*, *GAPDH*, *TBP*, *CC*, *EF-1-β*, *CYP*, *His3.3*, *TUB*, and *ACT* (Table 2). Similar as geNorm, NormFinder also identified *HELI*, *UBQ*, *CB*, and *RPL16* as the most four stable genes with the same ranking order, and *ACT* as the least stable one. There was only slight difference in the ranking of the mediate stable genes between the two programs.

The 12 candidate reference genes were further evaluated using comparative ∆Ct method. The results were similar to those from geNorm and NormFinder (Table 2). *HELI* was the most stable gene, and the other three (*RPL16*, *UBQ*, and *CB*) of the most four stable genes were the same as those identified using geNorm and NormFinder, although the ranking was different. From most to least stable, the order of the other 8 candidate genes was *CC*, *GAPDH*, *CYP*, *TBP*, *EF-1-β*, *TUB*, *His3.3*, and *ACT*. *ACT* was the least stable gene once again.

To get a final rank of the candidate genes, the results obtained from all the three algorithms were further analyzed using the method reported previously [48]. For each gene, the geometric mean of the ranking numbers generated from the three algorithms was calculated. Then all the genes were ranked again according to their geometric mean values, and gene with smaller value being more stable. As a result, *HELI*, *UBQ*, and *RPL16* were identified as the most stable genes for qRT-PCR analysis in different tissues of Yesso scallop. The final ranking orders of all the 12 genes were listed in Table 2.

### Expression profile of the candidate reference genes in scallop embryos/larvae

During different developmental stages of scallop embryos and larvae, the 12 candidate reference genes also presented variations in expression levels (Table S2, Figure 1B), with Ct values ranging from 16.2 to 35.2. Similar as in adult tissues, *CC* and *CB* were the most highly expressed genes in embryos/larvae, too, with a median Ct value of 17.8 and 18.1, respectively. The other 10 genes all had median Ct values greater than 20, and two (*UBQ* and *TBP*) of them showed Ct values greater than 30. In respect to gene expression variation among different developmental stages, *UBQ*, CB, CC, *His3.3* and *CYP* exhibited more narrow ranges of Ct dispersal than other genes, while *HELI*, *TUB*, and *ACT* showed greater variation (Figure 1B). The expression stability of the 12 candidate reference genes during scallop embryo/larva development was further evaluated with the three methods described above.

### Expression stability of the candidate reference genes in scallop embryos/larvae

For scallop embryos and larvae, geNorm, NormFinder, and the comparative ∆Ct method all identified CB, CC, and *His3.3* as the most stably expressed genes, and their ranking orders generated by the three programs were the same (Figure 2C, Table 2). Meanwhile, the least four stable genes revealed by the three programs were the same, too, but their ranking orders were slightly different except that of *HELI*. Both geNorm and comparative ∆Ct method showed that *TBP* was the least stable gene, while NormFinder identified *ACT* as the least. The ranking of other candidate genes provided by the three algorithms were more or less different. The comprehensive order of all the candidate genes was obtained, through calculating the geometric mean of the ranking numbers of each gene generated from the three algorithms. From most to least stable, the final ranking order was CB, CC, *His3.3*, *GAPDH*, *CYP*, *EF-1-β*, *RPL16*, *TUB*, *HELI*, *UBQ*, *ACT* and *TBP* (Table 2).

For the number of genes required for data normalization, geNorm analysis indicated that, the V3/4 value (0.160) was a little bit higher than the cut-off value 0.150. The V4/5 value was 0.123, thus the most four stable genes were required for accurate normalization (Figure 2D). According to the comprehensive rank of these genes, CB, CC, *His3.3*, and *CYP* could be recommended as the reference genes combination in qRT-PCR analysis with respect to Yesso scallop embryos/larvae

### Expression stability of the candidate reference genes among tissues and embryos/larvae

To find out a suitable reference genes combination which could be used for both tissues and embryos/larvae, the expression stability of the 12 candidate reference genes were assessed in all the samples together. As shown in Table S3, the three programs presented similar evaluation results, including the same most stable gene (*RPL16*) and the least stable gene (*ACT*). But no optimal gene combination could be provided. All the 10 Vn/Vn+1 values for the 12 candidate genes generated by geNorm were much higher than the suggested cut-off value 0.150 (Figure S1). Thus the number of genes required for reliable normalization could not be given. Meanwhile, the stability values (M) calculated by geNorm for most of the candidate genes were higher than the cut-off value 1.50, and only those of three genes were a little bit lower than 1.50. Therefore, the adoption of a specific combination of reference genes is recommended for each of the two sample sets (scallop tissues, and embryos/larvae).

## Discussion

As a powerful tool for detecting differentially expressed genes, qRT-PCR has been frequently used in bivalve to understand the biological processes of this large group of animals. For reliable and accurate qRT-PCR analysis, normalization with suitable reference genes is vital, when studying biologically related differences among distinct samples, such as from different tissues or developmental stages. In the present study, orthologs of 12 commonly used reference genes were selected from the transcriptome dataset of Yesso scallop, and their expression stabilities among adult tissues and embryos/larvae were evaluated.

For optimal reference gene selection, qRT-PCR data of the 12 candidate reference genes were analyzed using three statistical algorithms, geNorm, NormFinder, and the comparative ∆Ct method, which all were commonly used in reference gene evaluation [18,19,49,50]. Relatively consistent results were presented by the three programs, especially for the ranking of the most and the least stable reference genes. Among scallop tissues, both geNorm and NormFinder analysis showed that the order of the most four stable genes were *HELI*, *UBQ*, *CB*, and *RPL16*. The comparative ∆Ct method also identified these four genes as the most stable ones, and *HELI* ranked at the 1st place, while from the 2nd to the 4th, the order was *RPL16*, *UBQ*, and *CB*. The slight difference of gene ranking among the three programs was expected as their statistical algorithms are distinct [51-53]. *ACT* was revealed to be the least stable gene by all the three statistical approaches, and both geNorm and the comparative ∆Ct method gave the same ranking results for the least four ones. These least stable genes are not suitable for use in qRT-PCR analysis on different tissues in Yesso scallop. Based on the final comprehensive ranking and the number of genes required for optimal normalization, *HELI*, *UBQ*, and *RPL16* were recommended as the appropriate reference gene combination in gene expression analysis on different tissues of Yesso scallop. With respect to reference genes for scallop embryos/larvae, the three programs generated similar assessment results, too. CB, CC, and *His3.3* were listed as the 1st to 3rd stable genes by all the three algorithms. Also, the three approaches showed the same four genes as the least stable, although the ranking order was different. Finally, four genes including CB, CC, *His3.3*, and *GAPDH* were the suggested reference genes in qRT-PCR analysis for Yesso scallop embryos/larvae, while *ACT, TBP*, and *UBQ* should be avoid as control genes.

A literature analysis showed that, in 44 randomly selected publications describing target gene expression of scallops using qRT-PCR, the most frequently used reference gene is *ACT* (40 articles), followed by *GADPH* (2 articles) and *EF-1-β* (2 articles). However, their suitability as normalization factors for the corresponding samples was not evaluated. In this study, for both different tissues and different embryo/larva stages of scallop, the commonly used reference gene *ACT* was among the least stably expressed genes, while *EF-1-β* was mediate stable. As for *GADPH*, it was ranked as the 4th most stable gene and suggested to be used as reference gene for different embryo/larva stages, while in different tissues, it was mediate stable. So, for the sample sets we examined in this study, the currently used reference genes in scallops, especially *ACT* and *EF-1-β*, were less stable than those we recommended.

In addition, as an intention to find the generally appropriate reference genes for both scallop tissues and embryos/larvae, we analyzed all the expression data together. Although the three programs presented similar evaluation results (Table S3), no optimal gene combination could be provided, mainly for that no Vn/Vn+1 value was less than the suggested cut-off value 0.150 (Figure S1). The number of genes required for reliable normalization could not be given. Therefore, a specific combination of reference genes is recommended for scallop tissues and embryos/larvae, respectively. Similar results were reported in other studies, such as mammal [54] and plants [55-57], which also suggested that, for different set of experimental samples in a species, different suitable reference genes should be used for accurate qRT-PCR analysis.

In conclusion, we reported the first systematic analysis for the selection of superior reference genes used in qRT-PCR normalization with respect to normal tissues and embryo/larva stages in scallops. Through evaluating the expression stabilities of 12 candidate genes in 36 tissue samples and 15 embryo/larva samples from Yesso scallops, a specific combination of reference genes was recommended for each of the two sample sets. These reference genes will be useful for the identification of genes related to biological processes in Yesso scallop, and also in the reference gene selection for other scallop or bivalve species.

## Supporting Information

Table S1 **qRT-PCR Ct values for the 12 candidate reference genes obtained in Yesso scallop tissues.**
(DOC)

Table S2 **qRT-PCR Ct values for the 12 candidate reference genes obtained in Yesso scallop embryos/larvae.**
(DOC)

Table S3 **Ranks of the 12 candidate genes considering tissues and embryos/larvae together.**
(DOC)

Figure S1 **Pairwise variation analysis by geNorm to determine the number of reference genes required for qRT-PCR normalization when considering tissues and embryos/larvae together.**
(TIF)
