# Supplementary material for: Identification of Reference Genes for qRT-PCR Analysis in Yesso Scallop Patinopecten yessoensis
Source: PLoS One. 2013 Sep 19;8(9):e75609. doi: 10.1371/journal.pone.0075609 (PMC3777977; doi:10.1371/journal.pone.0075609)
Supplement: Table S1 — qRT-PCR Ct values for the 12 candidate reference genes obtained in Yesso scallop tissues. (DOC) [file pone.0075609.s001.doc]

**Table S1. qRT-PCR Ct values for the 12 candidate reference genes** **obtained in Yesso scallop tissues.**

|  | **Mantle** | **Gill** | **Gonad** | **Kidney** | **Striated muscle** | **Digestive gland** |
| --- | --- | --- | --- | --- | --- | --- |
| ACT | 18.7±1.9 | 21.2±0.6 | 19.5±0.8 | 23.6±1.8 | 14.6±0.2 | 22.5±1.6 |
| GAPDH | 24.8±2.0 | 22.0±0.4 | 21.2±1.6 | 22.4±1.8 | 19.3±0.7 | 22.0±0.7 |
| CC | 21.4±2.9 | 17.1±0.5 | 17.1±0.3 | 17.7±1.6 | 19.3±2.3 | 17.6±0.3 |
| CB | 19.8±1.0 | 17.6±0.4 | 17.7±0.4 | 18.4±1.5 | 19.8±2.2 | 18.1±0.3 |
| EF-1-β | 23.1±3.0 | 22.2±1.4 | 21.5±2.7 | 22.8±1.6 | 22.4±1.1 | 20.9±0.5 |
| UBQ | 28.6±0.9 | 27.1±1.2 | 27.4±1.9 | 28.6±3.1 | 29.0±1.4 | 27.5±0.3 |
| TBP | 31.1±0.5 | 28.4±0.8 | 28.4±2.1 | 30.1±3.6 | 31.0±1.4 | 27.7±0.7 |
| RPL16 | 20.9±0.4 | 19.4±0.6 | 20.2±1.9 | 21.2±0.9 | 21.2±1.1 | 19.5±0.4 |
| HELI | 27.4±0.9 | 25.6±1.0 | 25.5±2.1 | 26.8±2.3 | 26.6±1.7 | 25.9±0.4 |
| TUB | 24.6±1.0 | 27.1±0.9 | 21.8±3.3 | 22.8±1.4 | 24.7±2.3 | 27.3±0.6 |
| CYP | 26.7±1.6 | 22.9±0.8 | 24.1±1.7 | 25.8±1.1 | 25.2±1.9 | 22.8±0.9 |
| His3.3 | 26.8±4.2 | 23.1±4.4 | 21.9±1.1 | 21.8±1.3 | 23.6±0.7 | 21.1±0.5 |
