# Supplementary material for: Identification of Reference Genes for qRT-PCR Analysis in Yesso Scallop Patinopecten yessoensis
Source: PLoS One. 2013 Sep 19;8(9):e75609. doi: 10.1371/journal.pone.0075609 (PMC3777977; doi:10.1371/journal.pone.0075609)
Supplement: Table S2 — qRT-PCR Ct values for the 12 candidate reference genes obtained in Yesso scallop embryos/larvae. (DOC) [file pone.0075609.s002.doc]

**Table S2. qRT-PCR Ct values for the 12 candidate reference genes obtained in Yesso scallop embryos/larvae.**

|  | **Fertilized eggs** | **Blustulae** | **Gastrulae** | **Trochophore larvae** | **D-shaped larvae** |
| --- | --- | --- | --- | --- | --- |
| ACT | 26.6±1.6 | 31.3±0.4 | 31.6±0.2 | 24.2±0.1 | 29.3±0.2 |
| GAPDH | 25.9±0.1 | 32.0±0.2 | 28.9±0.1 | 27.1±0.1 | 27.9±0.1 |
| CC | 17.1±0.1 | 21.7±0.1 | 18.5±0.1 | 16.2±0.1 | 17.6±0.1 |
| CB | 17.4±0.1 | 22.0±0.1 | 18.9±0.1 | 16.6±0.1 | 18.1±0.1 |
| EF-1-β | 22.9±0.1 | 28.0±0.6 | 25.5±0.1 | 23.0±0.1 | 21.9±0.1 |
| UBQ | 31.3±0.4 | 34.4±0 | 35.1±0.4 | 31.7±0.3 | 35.2±0.8 |
| TBP | 33.3±1.0 | 34.0±0.6 | 33.0±0.3 | 28.9±0.2 | 27.8±0.1 |
| RPL16 | 22.0±0.1 | 26.6±0.1 | 24.0±0.1 | 21.2±0.1 | 20.1±0.1 |
| HELI | 30.6±0.6 | 33.3±1.6 | 29.6±0.3 | 27.5±0.1 | 29.4±0.7 |
| TUB | 26.2±0.1 | 32.2±0.1 | 28.0±0.1 | 24.3±0.1 | 27.1±0.1 |
| CYP | 22.4±0.1 | 28.1±0.1 | 24.7±0.1 | 23.4±0.1 | 22.5±0.1 |
| His3.3 | 18.8±0.1 | 24.0±0.1 | 20.6±0.1 | 19.1±0.1 | 18.4±0.1 |
