# Supplementary material for: Identification of Reference Genes for qRT-PCR Analysis in Yesso Scallop Patinopecten yessoensis
Source: PLoS One. 2013 Sep 19;8(9):e75609. doi: 10.1371/journal.pone.0075609 (PMC3777977; doi:10.1371/journal.pone.0075609)
Supplement: Table S3 — Ranks of the 12 candidate genes considering tissues and embryos/larvae together. (DOC) [file pone.0075609.s003.doc]

**Table S3. Ranks of the 12 candidate genes considering tissues and embryos/larvae together.**

| **Rank** | **geNorm** | **NormFinder** | **Comparative ΔCt method** | **Comprehensive**  **rank** |
| --- | --- | --- | --- | --- |
| 1 | RPL16 (1.343) | RPL16 (0.199) | RPL16 (1.856) | RPL16 |
| 2 | HELI (1.343) | EF-1-β(0.378) | HELI (2.082) | HELI |
| 3 | TBP (1.492) | TUB (0.390) | EF-1-β(2.095) | EF-1-β |
| 4 | CB (1.693) | TBP (0.567) | CYP (2.099) | TBP |
| 5 | CYP (1.782) | HELI (0.629) | CB (2.288) | CB |
| 6 | EF-1-β ( 1.855) | CB (0.972) | TBP (2.440) | CYP |
| 7 | CC (1.924) | CC (1.102) | CC (2.461) | TUB |
| 8 | UBQ (2.101) | CYP (1.192) | GAPDH (2.487) | CC |
| 9 | TUB (2.259) | UBQ (1.322) | TUB (2.487) | UBQ |
| 10 | GAPDH (2.421) | GAPDH (1.590) | UBQ (2.690) | GAPDH |
| 11 | His3.3 (2.683) | His3.3 (2.183) | His3.3 (3.013) | His3.3 |
| 12 | ACT (3.026) | ACT (2.575) | ACT (3.369) | ACT |

Note: The candidate reference genes were ordered from the most to the least stable, based on their stability values (in brackets) calculated by different algorithms. Lower stability value means more stable expression. The comprehensive rank for each gene was based on the geometric mean of its ranks from geNorm, NormFinder, and comparative ΔCt method, and gene with smaller value of geometric mean was ranked as more stable.
