# Supplementary figures and images for: Identification of Reference Genes for qRT-PCR Analysis in Yesso Scallop Patinopecten yessoensis
Source: PLoS One. 2013 Sep 19;8(9):e75609. doi: 10.1371/journal.pone.0075609 (PMC3777977; doi:10.1371/journal.pone.0075609)

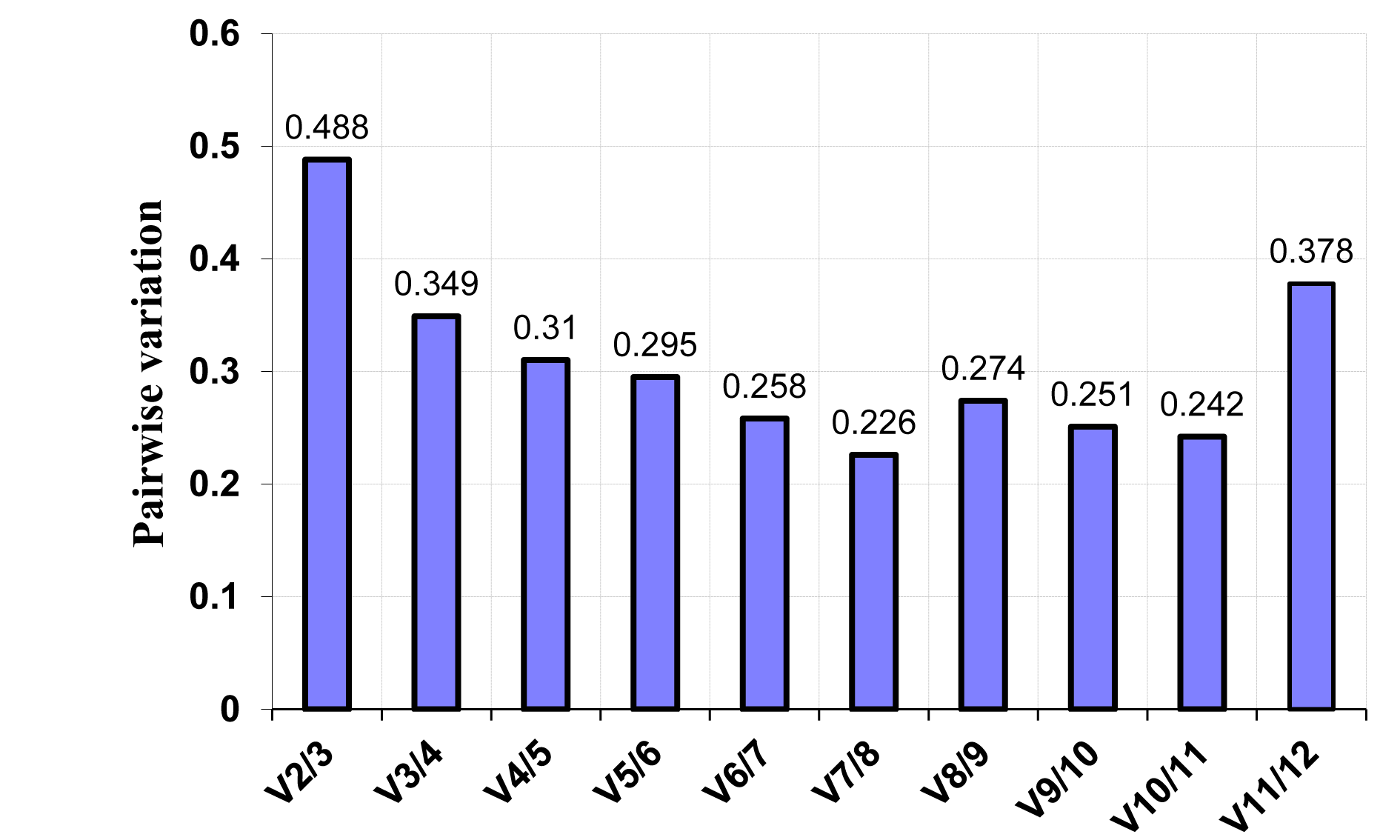

Supplement: Figure S1 — Pairwise variation analysis by geNorm to determine the number of reference genes required for qRT-PCR normalization when considering tissues and embryos/larvae together. (TIF) [file pone.0075609.s004.tif]
